# Supplementary material for: Renoprotective and haemodynamic effects of adiponectin and peroxisome proliferator-activated receptor agonist, pioglitazone, in renal vasculature of diabetic Spontaneously hypertensive rats
Source: PLoS One. 2020 Nov 10;15(11):e0229803. doi: 10.1371/journal.pone.0229803 (PMC7654782; doi:10.1371/journal.pone.0229803)
Supplement: S1 Table — (DOCX) [file pone.0229803.s001.docx]

| Parameters | Groups | Days of Observation | | | Day 28 |
| --- | --- | --- | --- | --- | --- |
|  |  | Day 0 | Day 8 | Day 21 |  |
| Body Weight (g) | WKY | 1. 245 2. 250 3. 240 4. 245 5. 240 6. 250 | 1. 250 2. 255 3. 245 4. 240 5. 250 6. 260 | 1. 275 2. 279 3. 271 4. 270 5. 275 6. 280 | 1. 289 2. 300 3. 280 4. 280 5. 290 6. 295 |
|  | SHR | 1. 242 2. 245 3. 239 4. 238 5. 245 6. 240 | 1. 248 2. 245 3. 240 4. 255 5. 255 6. 245 | 1. 267 2. 275 3. 260 4. 258 5. 278 6. 264 | 1. 284 2. 293 3. 270 4. 280 5. 284 6. 295 |
|  | SHR+STZ | 1. 245 2. 242 3. 248 4. 240 5. 246 6. 247 | 1. 200 2. 205 3. 198 4. 202 5. 204 6. 191 | 1. 208 2. 210 3. 200 4. 213 5. 217 6. 200 | 1. 209 2. 215 3. 197 4. 210 5. 223 6. 197 |
|  | SHR+STZ+Pio | 1. 254 2. 260 3. 248 4. 248 5. 252 6. 262 | 1. 205 2. 209 3. 201 4. 201 5. 205 6. 211 | 1. 207 2. 214 3. 200 4. 200 5. 207 6. 217 | 1. 217 2. 222 3. 212 4. 212 5. 221 6. 223 |
|  | SHR+STZ+Adp | 1. 252 2. 256 3. 248 4. 248 5. 252 6. 258 | 1. 213 2. 220 3. 206 4. 206 5. 213 6. 223 | 1. 215 2. 224 3. 206 4. 206 5. 212 6. 227 | 1. 206 2. 210 3. 202 4. 202 5. 206 6. 212 |
|  | SHR+STZ+Adp+Pio | 1. 247 2. 252 3. 242 4. 242 5. 247 6. 254 | 1. 201 2. 210 3. 192 4. 192 5. 201 6. 214 | 1. 200 2. 210 3. 190 4. 190 5. 200 6. 214 | 1. 209 2. 214 3. 204 4. 204 5. 209 6. 216 |
| Water intake  (ml/d) | WKY | 1. 43 2. 44 3. 42 4. 42 5. 42 6. 44 | 1. 44 2. 46 3. 42 4. 42 5. 44 6. 47 | 1. 45 2. 48 3. 42 4. 42 5. 44 6. 4 | 1. 45 2. 47 3. 43 4. 43 5. 45 6. 48 |
|  | SHR | 1. 32 2. 34 3. 30 4. 30 5. 32 6. 35 | 1. 34 2. 36 3. 32 4. 32 5. 34 6. 37 | 1. 34 2. 37 3. 31 4. 31 5. 34 6. 38 | 1. 37 2. 41 3. 33 4. 33 5. 37 6. 43 |
|  | SHR+STZ | 1. 33 2. 35 3. 31 4. 31 5. 33 6. 36 | 1. 48 2. 51 3. 45 4. 45 5. 48 6. 52 | 1. 48 2. 50 3. 46 4. 46 5. 48 6. 51 | 1. 59 2. 62 3. 56 4. 56 5. 59 6. 63 |
|  | SHR+STZ+Pio | 1. 34 2. 36 3. 32 4. 32 5. 34 6. 37 | 1. 48 2. 51 3. 45 4. 45 5. 48 6. 52 | 1. 50 2. 52 3. 48 4. 48 5. 50 6. 53 | 1. 50 2. 53 3. 47 4. 47 5. 50 6. 54 |
|  | SHR+STZ+Adp | 1. 36 2. 38 3. 34 4. 34 5. 36 6. 39 | 1. 46 2. 49 3. 43 4. 43 5. 46 6. 50 | 1. 47 2. 49 3. 45 4. 45 5. 47 6. 50 | 1. 57 2. 60 3. 54 4. 54 5. 57 6. 61 |
|  | SHR+STZ+Adp+Pio | 1. 34 2. 36 3. 32 4. 32 5. 34 6. 37 | 1. 45 2. 48 3. 42 4. 42 5. 45 6. 49 | 1. 49 2. 51 3. 47 4. 47 5. 49 6. 52 | 1. 55 2. 58 3. 52 4. 52 5. 55 6. 59 |
|  |  |  |  |  |  |
|  |  |  |  |  |  |
| UFR (mL/min/100 g) | WKY | 1. 3.84 2. 4.28 3. 3.4 4. 3.4 5. 3.65 6. 4.45 | 1. 3.63 2. 3.84 3. 3.42 4. 3.42 5. 3.60 6. 3.93 | 1. 3.92 2. 4.13 3. 3.71 4. 3.71 5. 3.86 6. 4.22 | 1. 3.95 2. 4.16 3. 3.74 4. 3.74 5. 3.87 6. 4.25 |
|  | SHR | 1. 3.10 2. 3.15 3. 3.05 4. 3.05 5. 3.10 6. 3.17 | 1. 2.98 2. 3.07 3. 2.89 4. 2.89 5. 2.98 6. 3.11 | 1. 2.94 2. 3.48 3. 2.4 4. 2.4 5. 2.73 6. 3.69 | 1. 2.93 2. 3.36 3. 2.5 4. 2.5 5. 2.80 6. 3.51 |
|  | SHR+STZ | 1. 3.09 2. 3.13 3. 3.05 4. 3.05 5. 3.09 6. 3.15 | 1. 12.35 2. 12.84 3. 11.8 4. 11.8 5. 12.21 6. 13.06 | 1. 12.79 2. 13.41 3. 12.17 4. 12.17 5. 12.56 6. 13.64 | 1. 13.18 2. 13.22 3. 13.14 4. 13.14 5. 13.15 6. 13.23 |
|  | SHR+STZ+Pio | 1. 3.07 2. 3.1 3. 3.04 4. 3.04 5. 3.07 6. 3.12 | 1. 13.58 2. 14.08 3. 13.08 4. 13.08 5. 13.43 6. 14.28 | 1. 13.57 2. 13.77 3. 13.37 4. 13.37 5. 13.52 6. 13.85 | 1. 13.58 2. 13.88 3. 13.28 4. 13.28 5. 13.49 6. 14.00 |
|  | SHR+STZ+Adp | 1. 3.06 2. 3.08 3. 3.04 4. 3.04 5. 3.06 6. 3.09 | 1. 13.28 2. 13.77 3. 12.79 4. 12.79 5. 13.10 6. 13.95 | 1. 13.35 2. 13.50 3. 13.20 4. 13.20 5. 13.30 6. 13.55 | 1. 16.25 2. 16.38 3. 16.12 4. 16.12 5. 16.25 6. 16.43 |
|  | SHR+STZ+Adp+Pio | 1. 3.09 2. 3.12 3. 3.06 4. 3.06 5. 3.09 6. 3.14 | 1. 13.57 2. 13.86 3. 13.28 4. 13.28 5. 13.50 6. 13.98 | 1. 13.58 2. 13.82 3. 13.34 4. 13.34 5. 13.52 6. 13.92 | 1. 20.28 2. 20.57 3. 20 4. 20 5. 20.15 6. 20.68 |
| Blood glucose (mg/dl) | WKY | 1. 89 2. 92 3. 86 4. 90 5. 85 6. 92 | 1. 88 2. 90 3. 85 4. 89 5. 87 6. 90 | 1. 86 2. 87 3. 85 4. 89 5. 83 6. 86 | 1. 88 2. 90 3. 87 4. 84 5. 85 6. 92 |
|  | SHR | 1. 91 2. 95 3. 89 4. 90 5. 94 6. 87 | 1. 90 2. 90 3. 87 4. 87 5. 91 6. 92 | 1. 88 2. 91 3. 84 4. 87 5. 86 6. 92 | 1. 89 2. 92 3. 89 4. 89 5. 89 6. 83 |
|  | SHR+STZ | 1. 90 2. 93 3. 91 4. 89 5. 89 6. 84 | 1. 460 2. 478 3. 480 4. 450 5. 456 6. 432 | 1. 471 2. 460 3. 462 4. 465 5. 475 6. 498 | 1. 489 2. 514 3. 465 4. 465 5. 475 6. 523 |
|  | SHR+STZ+Pio | 1. 90 2. 95 3. 84 4. 84 5. 94 6. 94 | 1. 471 2. 492 3. 452 4. 444 5. 470 6. 497 | 1. 477 2. 496 3. 457 4. 454 5. 478 6. 500 | 1. 474 2. 489 3. 459 4. 455 5. 476 6. 492 |
|  | SHR+STZ+Adp | 1. 88 2. 90 3. 86 4. 86 5. 89 6. 91 | 1. 465 2. 484 3. 446 4. 445 5. 461 6. 491 | 1. 462 2. 480 3. 444 4. 440 5. 462 6. 484 | 1. 484 2. 511 3. 457 4. 460 5. 470 6. 522 |
|  | SHR+STZ+Adp+Pio | 1. 86 2. 89 3. 83 4. 82 5. 87 6. 89 | 1. 479 2. 500 3. 458 4. 454 5. 478 6. 505 | 1. 486 2. 504 3. 468 4. 462 5. 490 6. 506 | 1. 480 2. 502 3. 458 4. 454 5. 478 6. 508 |
