# Supplementary material for: Renoprotective and haemodynamic effects of adiponectin and peroxisome proliferator-activated receptor agonist, pioglitazone, in renal vasculature of diabetic Spontaneously hypertensive rats
Source: PLoS One. 2020 Nov 10;15(11):e0229803. doi: 10.1371/journal.pone.0229803 (PMC7654782; doi:10.1371/journal.pone.0229803)
Supplement: S2 Table — (DOCX) [file pone.0229803.s002.docx]

| Parameters | Groups | Days of Observation | | | Day 28 |
| --- | --- | --- | --- | --- | --- |
|  |  | Day 0 | Day 8 | Day 21 |  |
| **Systolic blood pressure (mmHg)** | WKY | 1. 118 2. 127 3. 117 4. 112 5. 116 6. 119 | 1. 117 2. 115 3. 116 4. 118 5. 121 6. 115 | 1. 117 2. 119 3. 116 4. 118 5. 120 6. 114 | 1. 116 2. 119 3. 123 4. 124 5. 120 6. 118 |
|  | SHR | 1. 155 2. 166 3. 156 4. 161 5. 159 6. 158 | 1. 165 2. 166 3. 171 4. 160 5. 169 6. 153 | 1. 165 2. 166 3. 158 4. 161 5. 160 6. 164 | 1. 150 2. 148 3. 166 4. 157 5. 156 6. 165 |
|  | SHR+STZ | 1. 158 2. 165 3. 164 4. 169 5. 156 6. 157 | 1. 173 2. 170 3. 176 4. 168 5. 175 6. 177 | 1. 172 2. 175 3. 181 4. 174 5. 179 6. 182 | 1. 177 2. 176 3. 174 4. 180 5. 173 6. 170 |
|  | SHR+STZ+Pio | 1. 165 2. 166 3. 173 4. 156 5. 169 6. 161 | 1. 175 2. 180 3. 185 4. 174 5. 179 6. 181 | 1. 154 2. 155 3. 152 4. 153 5. 156 6. 160 | 1. 138 2. 143 3. 153 4. 156 5. 151 6. 147 |
|  | SHR+STZ+Adp | 1. 159 2. 166 3. 158 4. 161 5. 160 6. 168 | 1. 177 2. 176 3. 174 4. 172 5. 173 6. 178 | 1. 173 2. 170 3. 176 4. 171 5. 175 6. 179 | 1. 135 2. 136 3. 144 4. 143 5. 139 6. 133 |
|  | SHR+STZ+Adp+Pio | 1. 159 2. 166 3. 158 4. 165 5. 160 6. 171 | 1. 172 2. 175 3. 181 4. 174 5. 179 6. 182 | 1. 148 2. 155 3. 152 4. 153 5. 156 6. 160 | 1. 132 2. 130 3. 137 4. 139 5. 135 6. 131 |
| **Diastolic blood pressure(mmHg)** | WKY | 1. 75 2. 77 3. 78 4. 79 5. 84 6. 82 | 1. 90 2. 87 3. 78 4. 80 5. 84 6. 85 | 1. 80 2. 77 3. 78 4. 79 5. 84 6. 82 | 1. 91 2. 92 3. 81 4. 80 5. 83 6. 90 |
|  | SHR | 1. 110 2. 115 3. 119 4. 122 5. 124 6. 126 | 1. 107 2. 115 3. 113 4. 122 5. 125 6. 120 | 1. 106 2. 112 3. 98 4. 118 5. 105 6. 110 | 1. 112 2. 115 3. 120 4. 122 5. 124 6. 127 |
|  | SHR+STZ | 1. 112 2. 117 3. 113 4. 121 5. 119 6. 120 | 1. 116 2. 117 3. 119 4. 122 5. 121 6. 120 | 1. 116 2. 117 3. 119 4. 122 5. 123 6. 124 | 1. 115 2. 117 3. 113 4. 121 5. 119 6. 124 |
|  | SHR+STZ+Pio | 1. 112 2. 115 3. 113 4. 121 5. 117 6. 118 | 1. 115 2. 117 3. 119 4. 122 5. 121 6. 120 | 1. 109 2. 112 3. 100 4. 115 5. 108 6. 110 | 1. 109 2. 103 3. 98 4. 108 5. 108 6. 110 |
|  | SHR+STZ+Adp | 1. 111 2. 117 3. 113 4. 121 5. 119 6. 121 | 1. 114 2. 117 3. 119 4. 122 5. 123 6. 125 | 1. 115 2. 117 3. 113 4. 121 5. 119 6. 124 | 1. 94 2. 92 3. 95 4. 97 5. 98 6. 100 |
|  | SHR+STZ+Adp+Pio | 1. 115 2. 117 3. 113 4. 121 5. 119 6. 123 | 1. 118 2. 117 3. 119 4. 122 5. 121 6. 123 | 1. 106 2. 114 3. 111 4. 113 5. 115 6. 107 | 1. 97 2. 96 3. 99 4. 101 5. 95 6. 100 |
| **Mean arterial pressure (mmHg)** | WKY | 1. 84 2. 86 3. 98 4. 102 5. 82 6. 100 | 1. 92 2. 93 3. 96 4. 97 5. 100 6. 104 | 1. 87 2. 88 3. 90 4. 92 5. 96 6. 99 | 1. 90 2. 92 3. 96 4. 97 5. 103 6. 104 |
|  | SHR | 1. 129 2. 125 3. 130 4. 134 5. 135 6. 139 | 1. 130 2. 127 3. 132 4. 134 5. 135 6. 140 | 1. 120 2. 119 3. 124 4. 130 5. 131 6. 132 | 1. 127 2. 123 3. 130 4. 134 5. 135 6. 143 |
|  | SHR+STZ | 1. 127 2. 122 3. 130 4. 134 5. 135 6. 144 | 1. 131 2. 134 3. 132 4. 137 5. 142 6. 146 | 1. 137 2. 139 3. 142 4. 145 5. 148 6. 147 | 1. 136 2. 141 3. 143 4. 146 5. 148 6. 150 |
|  | SHR+STZ+Pio | 1. 127 2. 128 3. 130 4. 134 5. 135 6. 138 | 1. 133 2. 135 3. 138 4. 139 5. 144 6. 145 | 1. 118 2. 122 3. 124 4. 125 5. 127 6. 128 | 1. 112 2. 116 3. 120 4. 122 5. 124 6. 126 |
|  | SHR+STZ+Adp | 1. 128 2. 130 3. 132 4. 134 5. 132 6. 136 | 1. 132 2. 133 3. 135 4. 138 5. 142 6. 148 | 1. 131 2. 134 3. 132 4. 137 5. 142 6. 146 | 1. 98 2. 106 3. 110 4. 112 5. 114 6. 120 |
|  | SHR+STZ+Adp+Pio | 1. 130 2. 127 3. 132 4. 134 5. 135 6. 140 | 1. 133 2. 135 3. 138 4. 139 5. 144 6. 145 | 1. 120 2. 122 3. 124 4. 126 5. 128 6. 130 | 1. 98 2. 100 3. 102 4. 106 5. 108 6. 116 |
| **HR (beat/min)** | WKY | 1. 295 2. 306 3. 312 4. 316 5. 318 6. 325 | 1. 302 2. 306 3. 308 4. 310 5. 314 6. 316 | 1. 292 2. 304 3. 309 4. 310 5. 313 6. 326 | 1. 290 2. 299 3. 303 4. 304 5. 308 6. 314 |
|  | SHR | 1. 376 2. 380 3. 382 4. 384 5. 394 6. 400 | 1. 376 2. 380 3. 390 4. 394 5. 398 6. 402 | 1. 374 2. 386 3. 388 4. 390 5. 398 6. 416 | 1. 376 2. 380 3. 384 4. 392 5. 396 6. 406 |
|  | SHR+STZ | 1. 380 2. 382 3. 384 4. 386 5. 388 6. 402 | 1. 376 2. 388 3. 390 4. 392 5. 394 6. 424 | 1. 396 2. 398 3. 402 4. 403 5. 405 6. 408 | 1. 400 2. 405 3. 406 4. 407 5. 409 6. 415 |
|  | SHR+STZ+Pio | 1. 379 2. 386 3. 387 4. 388 5. 392 6. 396 | 1. 396 2. 398 3. 399 4. 400 5. 401 6. 406 | 1. 376 2. 378 3. 379 4. 380 5. 381 6. 386 | 1. 360 2. 365 3. 367 4. 368 5. 369 6. 373 |
|  | SHR+STZ+Adp | 1. 378 2. 381 3. 382 4. 383 5. 385 6. 389 | 1. 390 2. 396 3. 398 4. 399 5. 400 6. 405 | 1. 389 2. 393 3. 394 4. 395 5. 396 6. 403 | 1. 351 2. 354 3. 356 4. 357 5. 358 6. 360 |
|  | SHR+STZ+Adp+Pio | 1. 376 2. 386 3. 387 4. 388 5. 389 6. 396 | 1. 396 2. 401 3. 402 4. 403 5. 404 6. 412 | 1. 367 2. 375 3. 376 4. 377 5. 381 6. 386 | 1. 344 2. 349 3. 350 4. 351 5. 355 6. 357 |
