# Supplementary material for: Renoprotective and haemodynamic effects of adiponectin and peroxisome proliferator-activated receptor agonist, pioglitazone, in renal vasculature of diabetic Spontaneously hypertensive rats
Source: PLoS One. 2020 Nov 10;15(11):e0229803. doi: 10.1371/journal.pone.0229803 (PMC7654782; doi:10.1371/journal.pone.0229803)
Supplement: S3 Table — (PDF) [file pone.0229803.s003.pdf]

Parameters	Groups	Days of Observation			Day 28
		Day 0	Day 8	Day 21	
**Cr.Cl(ml/min/kg)**	WKY	1) 0.45<br>2) 0.46<br>3) 0.42<br>4) 0.47<br>5) 0.46<br>6) 0.45	1) 0.41<br>2) 0.43<br>3) 0.38<br>4) 0.41<br>5) 0.42<br>6) 0.43	1) 0.42<br>2) 0.42<br>3) 0.42<br>4) 0.42<br>5) 0.42<br>6) 0.42	1) 0.41<br>2) 0.43<br>3) 0.41<br>4) 0.38<br>5) 0.42<br>6) 0.43
	SHR	1) 0.30<br>2) 0.31<br>3) 0.27<br>4) 0.30<br>5) 0.30<br>6) 0.33	1) 0.33<br>2) 0.31<br>3) 0.33<br>4) 0.34<br>5) 0.33<br>6) 0.34	1) 0.32<br>2) 0.30<br>3) 0.31<br>4) 0.33<br>5) 0.33<br>6) 0.33	1) 0.33<br>2) 0.34<br>3) 0.33<br>4) 0.33<br>5) 0.34<br>6) 0.31
	SHR+STZ	1) 0.32<br>2) 0.33<br>3) 0.33<br>4) 0.33<br>5) 0.30<br>6) 0.31	1) 0.90<br>2) 0.95<br>3) 0.85<br>4) 0.91<br>5) 0.95<br>6) 0.84	1) 0.92<br>2) 0.99<br>3) 0.81<br>4) 0.95<br>5) 0.99<br>6) 0.85	1) 1.15<br>2) 1.26<br>3) 1.06<br>4) 1.18<br>5) 0.99<br>6) 1.26
	SHR+STZ+Pio	1) 0.31<br>2) 0.33<br>3) 0.33<br>4) 0.30<br>5) 0.28<br>6) 0.32	1) 0.94<br>2) 0.45<br>3) 1.40<br>4) 1.48<br>5) 0.99<br>6) 0.36	1) 0.95<br>2) 1.00<br>3) 0.90<br>4) 1.03<br>5) 0.92<br>6) 0.90	1) 1.45<br>2) 1.41<br>3) 1.46<br>4) 1.45<br>5) 1.45<br>6) 1.48
	SHR+STZ+Adp	1) 0.30<br>2) 0.30<br>3) 0.35<br>4) 0.20<br>5) 0.30<br>6) 0.35	1) 0.90<br>2) 0.94<br>3) 0.90<br>4) 0.87<br>5) 0.85<br>6) 0.96	1) 0.09<br>2) 0.05<br>3) 0.01<br>4) 0.09<br>5) 0.16<br>6) 0.19	1) 1.87<br>2) 1.98<br>3) 1.70<br>4) 1.70<br>5) 1.99<br>6) 1.99
	SHR+STZ+Adp+Pio	1) 0.31<br>2) 0.33<br>3) 0.33<br>4) 0.30<br>5) 0.28<br>6) 0.32	1) 0.93<br>2) 0.98<br>3) 0.88<br>4) 1.01<br>5) 0.90<br>6) 0.88	1) 0.96<br>2) 0.21<br>3) 1.43<br>4) 1.42<br>5) 1.43<br>6) 0.33	1) 0.94<br>2) 0.55<br>3) 2.50<br>4) 1.95<br>5) 2.88<br>6) 2.82
**Urinary Cr (mg/dl)**	WKY	1) 151.87<br>2) 153.75<br>3) 149.99<br>4) 149.99<br>5) 151.20<br>6) 154.43	1) 152.85<br>2) 155.74<br>3) 148.30<br>4) 152.44<br>5) 154.87<br>6) 155.77	1) 154.32<br>2)156.25<br>3)156.30<br>4)156.88<br>5)157.27<br>6)156.29	1) 157.05<br>2)157.75<br>3)157.55<br>4)158.63<br>5)156.31<br>6)155.01
	SHR	1) 99.34<br>2)101.99<br>3) 100.00<br>4) 98.49<br>5)99.01<br>6)97.20	1) 103.57<br>2) 105.03<br>3) 103.55<br>4) 100.97<br>5) 100.50<br>6)107.80	1) 104.23<br>2)106.39<br>3)104.05<br>4)100.15<br>5)105.75<br>6)104.81	1)102.39<br>2)105.90<br>3)98.01<br>4)102.00<br>5)104.02<br>6)102.02
	SHR+STZ	1) 100.38<br>2) 100.10<br>3) 103.00<br>4) 100.01	1) 77.81<br>2)80.45<br>3)73.49<br>4)83.15	1) 79.25<br>2)84.35<br>3)82.90<br>4)76.83	1) 81.74<br>2) 83.20<br>3)81.72<br>4)79.14

		5) 97.99<br>6) 100.80	5)75.10<br>6)76.85	5)75.10<br>6)77.06	5)78.67<br>6)85.97
	SHR+STZ+Pio	1) 102.38<br>2) 104.92<br>3) 99.84<br>4) 99.84 5)<br>101.45<br>6) 105.85	1) 79.36<br>2)81.94<br>3)76.78<br>4)76.78<br>5)78.41<br>6)82.89	1) 81.58<br>2)83.51<br>3)79.65<br>4)79.65<br>5)84.21<br>6)80.90	1) 86.23<br>2)83.10<br>3)83.10<br>4)89.36<br>5)90.51<br>6)85.10
	SHR+STZ+Adp	1) 101.87<br>2) 104.58<br>3) 99.16<br>4) 99.16<br>5) 100.88<br>6) 105.59	1) 77.39<br>2)80.06<br>3)74.72<br>4)74.72<br>5)81.04<br>6)76.41	1) 79.58<br>2)81.77<br>3)77.39<br>4)77.39<br>5)82.57<br>6)78.78	1) 93.57<br>2)95.24<br>3)91.90<br>4)91.90<br>5)95.81<br>6)93.00
	SHR+STZ+Adp+Pio	1) 93.57<br>2) 95.24<br>3) 91.90<br>4) 91.90<br>5) 95.81<br>6) 93.00	1) 80.23<br>2)82.72<br>3)77.74<br>4)77.74<br>5) 79.30<br>6) 83.63	1) 80.31<br>2)82.28<br>3)78.34<br>4)78.34<br>5)83.01<br>6)79.56	1) 104.58<br>2)106.98<br>3)102.18<br>4)102.18<br>5)107.89<br>6)103.65
**Urinary Na conc. (mmol/L)**	WKY	1) 137.88<br>2) 139.28<br>3) 136.32<br>4) 136.38<br>5) 139.38<br>6) 137.80	1) 139.18<br>2) 140.78<br>3) 137.68<br>4) 137.52<br>5) 139.10<br>6) 141.10	1) 140.28<br>2) 142.68<br>3) 137.82<br>4) 137.78<br>5) 142.58<br>6) 140.36	1) 141.22<br>2) 144.18<br>3) 138.22<br>4) 138.38<br>5) 144.16<br>6) 141.34
	SHR	1) 91.06<br>2) 93.64<br>3) 88.42<br>4) 88.38<br>5) 93.66<br>6) 91.02	1) 94.42<br>2) 96.48<br>3) 92.12<br>4) 92.18<br>5) 96.58<br>6) 94.28	1) 98.63<br>2) 103.94<br>3) 93.32<br>4) 93.22<br>5) 103.94<br>6) 98.74	1) 95.67<br>2) 99.26<br>3) 92.08<br>4) 92.08<br>5) 95.67<br>6) 99.26
	SHR+STZ	1) 95.61<br>2) 98.14<br>3) 93.08<br>4) 93.05<br>5) 95.60<br>6) 98.18	1) 117.88<br>2) 115.30<br>3) 120.46<br>4) 120.58<br>5) 118.00<br>6) 115.10	1) 121.28<br>2) 124.00<br>3) 118.60<br>4) 118.60<br>5) 121.20<br>6) 124.19	1) 120.96<br>2) 118.18<br>3) 123.55<br>4) 123.55<br>5) 121.12<br>6) 118.48
	SHR+STZ+Pio	1) 95.31<br>2) 97.88<br>3) 92.86<br>4) 92.82<br>5) 97.94<br>6) 95.42	1) 117.36<br>2) 120.78<br>3) 113.98<br>4) 114.00<br>5) 117.18<br>6) 120.94	1) 120.28<br>2) 124.18<br>3) 116.78<br>4) 116.80<br>5) 124.00<br>6) 120.20	1) 123.56<br>2) 126.00<br>3) 121.18<br>4) 121.24<br>5) 126.10<br>6) 123.56
	SHR+STZ+Adp	1) 96.31<br>2) 99.18<br>3) 93.30<br>4) 93.05<br>5) 96.10<br>6) 99.95	1) 119.31<br>2) 121.78<br>3) 116.84<br>4) 119.31<br>5) 122.32<br>6) 116.27	1) 117.37<br>2) 121.68<br>3) 113.06<br>4) 113.05<br>5) 115.80<br>6) 123.26	1) 131.89<br>2) 134.60<br>3) 129.18<br>4) 129.18<br>5) 130.90<br>6) 135.60

	SHR+STZ+Adp+Pio	1) 96.31<br>2) 98.29<br>3) 94.33<br>4) 94.33<br>5) 95.57<br>6) 99.02	1) 115.28<br>2) 117.85<br>3) 112.71<br>4) 112.71<br>5) 114.34<br>6) 118.79	1) 123.25<br>2) 125.68<br>3) 120.82<br>4) 120.82<br>5) 122.36<br>6) 126.57	1) 142.37<br>2) 145.52<br>3) 139.22<br>4) 139.22<br>5) 141.23<br>6) 146.66
**$U_{Na}V$ (mmol/hr/100g)**	WKY	1) 0.22<br>2) 0.23<br>3) 0.23<br>4) 0.23<br>5) 0.21<br>6) 0.21	1) 0.23<br>2) 0.21<br>3) 0.22<br>4) 0.24<br>5) 0.24<br>6) 0.24	1) 0.23<br>2) 0.21<br>3) 0.22<br>4) 0.24<br>5) 0.24<br>6) 0.24	1) 0.24<br>2) 0.24<br>3) 0.24<br>4) 0.24<br>5) 0.24<br>6) 0.24
	SHR	1) 0.01<br>2) 0.01<br>3) 0.06<br>4) 0.09,<br>5) 0.01<br>6) 0.06	1) 0.01<br>2) 0.05<br>3) 0.01<br>4) 0.02<br>5) 0.04<br>6) 0.07	1) 0.01<br>2) 0.04<br>3) 0.03<br>4) 0.03,<br>5) 0.04<br>6) 0.01	1) 0.14<br>2) 0.14<br>3) 0.19<br>4) 0.04<br>5) 0.14<br>6) 0.19
	SHR+STZ	1) 0.01<br>2) 0.04<br>3) 0.03<br>4) 0.03,<br>5) 0.04<br>6) 0.01	1) 0.06<br>2) 0.09<br>3) 0.08<br>4) 0.08<br>5) 0.01<br>6) 0.06	1) 0.08<br>2) 0.10<br>3) 0.10<br>4) 0.07<br>5) 0.05<br>6) 0.09	1) 0.10<br>2) 0.10<br>3) 0.14<br>4) 0.16<br>5) 0.05<br>6) 0.07
	SHR+STZ+Pio	1) 0.01<br>2) 0.04<br>3) 0.03<br>4) 0.03,<br>5) 0.04<br>6) 0.01	1) 0.07<br>2) 0.02<br>3) 0.04<br>4) 0.07<br>5) 0.11<br>6) 0.13	1) 0.11<br>2) 0.10<br>3) 0.12<br>4) 0.08<br>5) 0.13<br>6) 0.13	1) 0.14<br>2) 0.21<br>3) 0.17<br>4) 0.21<br>5) 0.03<br>6) 0.07
	SHR+STZ+Adp	1) 0.01<br>2) 0.00<br>3) 0.00<br>4) 0.02<br>5) 0.02<br>6) 0.02	1) 0.06<br>2) 0.08<br>3) 0.07<br>4) 0.08<br>5) 0.05<br>6) 0.03	1) 0.07<br>2) 0.09<br>3) 0.09<br>4) 0.10<br>5) 0.02<br>6) 0.07	1) 0.17<br>2) 0.23<br>3) 0.10<br>4) 0.19<br>5) 0.24<br>6) 0.09
	SHR+STZ+Adp+Pio	1) 0.01<br>2) 0.01<br>3) 0.05<br>4) 0.02<br>5) 0.04<br>6) 0.07	1) 0.07<br>2) 0.10<br>3) 0.02<br>4) 0.07<br>5) 0.09<br>6) 0.09	1) 0.11<br>2) 0.03<br>3) 0.18<br>4) 0.13<br>5) 0.04<br>6) 0.17	1) 0.23<br>2) 0.23<br>3) 0.23<br>4) 0.28<br>5) 0.28<br>6) 0.13
**FENa ((%)**	WKY	1) 0.50<br>2) 0.51<br>3) 0.52<br>4) 0.52<br>5) 0.49	1) 0.53<br>2) 0.54<br>3) 0.52<br>4) 0.52<br>5) 0.54	1) 0.59<br>2) 0.60<br>3) 0.60<br>4) 0.60<br>5) 0.58	1) 0.54<br>2) 0.49<br>3) 0.54<br>4) 0.57<br>5) 0.56

		6) 0.47	6) 0.54	6) 0.58	6) 0.56
	SHR	1) 0.35<br>2) 0.37<br>3) 0.37<br>4) 0.38<br>5) 0.35<br>6) 0.30	1) 0.37<br>2) 0.32<br>3) 0.39<br>4) 0.39<br>5) 0.40<br>6) 0.37	1) 0.37<br>2) 0.38<br>3) 0.39<br>4) 0.39<br>5) 0.36<br>6) 0.34	1) 0.34<br>2) 0.33<br>3) 0.33<br>4) 0.35<br>5) 0.35<br>6) 0.35
	SHR+STZ	1) 0.33<br>2) 0.34<br>3) 0.35<br>4) 0.32<br>5) 0.35<br>6) 0.30	1) 0.79<br>2) 0.79<br>3) 0.74<br>4) 0.81<br>5) 0.81<br>6) 0.84	1) 0.99<br>2) 0.89<br>3) 1.04<br>4) 1.04<br>5) 0.99<br>6) 0.99	1) 1.05<br>2) 1.00<br>3) 1.02<br>4) 1.05<br>5) 1.09<br>6) 1.11
	SHR+STZ+Pio	1) 0.33<br>2) 0.28<br>3) 0.30<br>4) 0.33<br>5) 0.37<br>6) 0.39	1) 0.99<br>2) 0.99<br>3) 1.04<br>4) 1.04<br>5) 0.89<br>6) 0.99	1) 1.10<br>2) 1.10<br>3) 1.05<br>4) 1.12<br>5) 1.15<br>6) 1.12	1) 1.11<br>2) 1.01<br>3) 1.11<br>4) 1.11<br>5) 1.16<br>6) 1.16
	SHR+STZ+Adp	1) 0.32<br>2) 0.32<br>3) 0.29<br>4) 0.27<br>5) 0.36<br>6) 0.38	1) 0.84<br>2) 0.84<br>3) 0.79<br>4) 0.81<br>5) 0.90<br>6) 0.88	1) 0.86<br>2) 0.83<br>3) 0.88<br>4) 0.85<br>5) 0.88<br>6) 0.87	1) 1.12<br>2) 1.12<br>3) 1.14<br>4) 1.14<br>5) 1.07<br>6) 1.17
	SHR+STZ+Adp+Pio	1) 0.33<br>2) 0.33<br>3) 0.33<br>4) 0.23<br>5) 0.38<br>6) 0.38	1) 0.10<br>2) 0.05<br>3) 0.10<br>4) 0.07<br>5) 0.14<br>6) 0.16	1) 1.11<br>2) 1.37<br>3) 0.77<br>4) 1.39<br>5) 1.15<br>6) 0.87	1) 1.23<br>2) 1.15<br>3) 1.30<br>4) 1.25<br>5) 1.16<br>6) 1.29
**Na:K**	WKY	1) 3.00<br>2) 3.08<br>3) 2.92<br>4) 2.92<br>5) 3.00<br>6) 3.10	1) 3.09<br>2) 3.16<br>3) 3.02<br>4) 3.02<br>5) 3.09<br>6) 3.18	1) 3.02<br>2) 3.06<br>3) 2.98<br>4) 2.98<br>5) 3.06<br>6) 3.02	1) 3.02<br>2) 3.17<br>3) 2.91<br>4) 2.91<br>5) 3.17<br>6) 3.08
	SHR	1) 1.34<br>2) 1.45<br>3) 1.20<br>4) 1.18<br>5) 1.55<br>6) 1.40	1) 1.40<br>2) 1.54<br>3) 1.12<br>4) 1.10<br>5) 1.60<br>6) 1.44	1) 1.32<br>2) 1.5<br>3) 1.14<br>4) 1.14<br>5) 1.56<br>6) 1.28	1) 1.42<br>2) 1.73<br>3) 1.12<br>4) 1.76<br>5) 1.40<br>6) 1.00
	SHR+STZ	1) 1.39<br>2) 1.62<br>3) 1.16<br>4) 1.14<br>5) 1.64<br>6) 1.41	1) 0.36<br>2) 0.51<br>3) 0.21<br>4) 0.21<br>5) 0.54<br>6) 0.34	1) 0.32<br>2) 0.57<br>3) 0.44<br>4) 0.64<br>5) 0.58<br>6) 0.26	1) 0.30<br>2) 0.53<br>3) 0.48<br>4) 0.63<br>5) 0.55<br>6) 0.37
	SHR+STZ+Pio	1) 1.41<br>2) 1.66<br>3) 1.14	1) 0.35<br>2) 0.53<br>3) 0.17	1) 0.66<br>2) 0.92<br>3) 0.35	1) 0.83<br>2) 0.52<br>3) 1.15

		4) 1.13<br>5) 1.69<br>6) 1.44	4) 0.17<br>5) 0.30<br>6) 0.58	4) 0.35<br>5) 0.95<br>6) 0.70	4) 1.20<br>5)0.80<br>6) 0.48
	SHR+STZ+Adp	1) 1.39<br>2) 1.64<br>3) 1.14<br>4) 1.16<br>5) 1.70<br>6) 1.30	1) 0.35<br>2) 0.54<br>3) 0.16<br>4) 0.12<br>5) 0.58<br>6) 0.36	1) 0.32<br>2) 0.16<br>3) 0.48<br>4) 0.48<br>5) 0.36<br>6) 0.10	1) 1.29<br>2) 1.41<br>3) 1.17<br>4) 1.16<br>5) 1.40<br>6) 1.35
	SHR+STZ+Adp+Pio	1) 1.38<br>2) 1.57<br>3) 1.12<br>4) 1.19<br>5) 1.58<br>6) 1.46	1) 0.36<br>2) 0.62<br>3) 0.08<br>4) 0.08<br>5) 0.60<br>6) 0.44	1) 0.7<br>2) 0.37<br>3) 1.03<br>4) 1.06<br>5) 0.30<br>6) 0.78	1) 1.61<br>2) 1.92<br>3) 1.3<br>4) 1.3<br>5) 1.52<br>6)2.02
**Urinary K Conc. (mmol/L)**	WKY	43.6<br>44.2<br>44.5<br>45<br>45.5<br>46.5	48.8<br>46.8<br>46<br>45.6<br>44.8<br>43	46.1<br>44.4<br>43.8<br>43.4<br>42.8<br>40.5	46.2<br>44.8<br>44<br>43.8<br>43.2<br>42.6
	SHR	58.3<br>56.7<br>56<br>55.4<br>54.8<br>52.9	56.5<br>56.8<br>56<br>54.8<br>53.6<br>53.5	58.9<br>56.8<br>56.4<br>56<br>55.6<br>53.5	58.8<br>58<br>57<br>56.7<br>56.1<br>55.2
	SHR+STZ	58.2<br>56.8<br>56<br>55.2<br>55<br>54.2	173<br>170<br>168<br>167<br>161<br>159	197<br>194<br>193<br>190<br>188<br>186	222.2<br>218<br>214.5<br>211.5<br>208.3<br>204.8
	SHR+STZ+Pio	58.3<br>57.2<br>56.2<br>55.4<br>55.2<br>54.7	188.5<br>185<br>180<br>178<br>176<br>174	155<br>153<br>151<br>150<br>147<br>145	116.5<br>112<br>110<br>108<br>106<br>103
	SHR+STZ+Adp	58.5<br>57<br>56<br>55<br>54<br>53.5	190<br>188<br>187<br>185<br>183<br>180	206<br>202<br>201<br>200<br>196<br>190	123<br>118<br>116<br>115<br>112<br>107
	SHR+STZ+Adp+Pio	58.3<br>57.2<br>56.2<br>55.4<br>55.2<br>54.7	195<br>192<br>191<br>190<br>188<br>185	151<br>148<br>146<br>147<br>145<br>140	99<br>95<br>94<br>93<br>96<br>89
