# Supplementary material for: Renoprotective and haemodynamic effects of adiponectin and peroxisome proliferator-activated receptor agonist, pioglitazone, in renal vasculature of diabetic Spontaneously hypertensive rats
Source: PLoS One. 2020 Nov 10;15(11):e0229803. doi: 10.1371/journal.pone.0229803 (PMC7654782; doi:10.1371/journal.pone.0229803)
Supplement: S1 File — (DOCX) [file pone.0229803.s004.docx]

| **Parameters** | **Groups** |  |
| --- | --- | --- |
|  | | **Values /rat** |
| **Adiponectin (µg/ml)** | WKY | 1. 18 2. 19 3. 20.5 4. 19.5 5. 20.7 6. 21.6 |
|  | SHR | 1. 7.1 2. 6.6 3. 7.2 4. 7.6 5. 7.9 6. 7.1 |
|  | SHR+STZ | 1. 5.9 2. 5.3 3. 5.7 4. 5.1 5. 5.1 6. 5.9 |
|  | SHR+STZ+Pio | 1. 8.99 2. 9.07 3. 9.11 4. 8.88 5. 9.15 6. 9.09 |
|  | SHR+STZ+Adp | 1. 11.4 2. 11.3 3. 11.2 4. 12.4 5. 11.8 6. 11.9 |
|  | SHR+STZ+Adp+Pio | 1. 13.4 2. 13.5 3. 13.9 4. 14.1 5. 13.8 6. 13.5 |
