# Supplementary material for: Renoprotective and haemodynamic effects of adiponectin and peroxisome proliferator-activated receptor agonist, pioglitazone, in renal vasculature of diabetic Spontaneously hypertensive rats
Source: PLoS One. 2020 Nov 10;15(11):e0229803. doi: 10.1371/journal.pone.0229803 (PMC7654782; doi:10.1371/journal.pone.0229803)
Supplement: S2 File — (DOCX) [file pone.0229803.s005.docx]

| **Parameters** | **Groups** |  |
| --- | --- | --- |
|  | | **Values /rat** |
| **Renal cortical blood perfusion (BPU)** | WKY | 1. 244 2. 247 3. 251 4. 255 5. 240 6. 237 |
|  | SHR | 1. 165 2. 168 3. 167 4. 171 5. 174 6. 166 |
|  | SHR+STZ | 1. 135 2. 129 3. 151 4. 133 5. 139 6. 144 |
|  | SHR+STZ+Pio | 1. 164 2. 167 3. 166 4. 171 5. 172 6. 181 |
|  | SHR+STZ+Adp | 1. 188 2. 191 3. 187 4. 194 5. 187 6. 200 |
|  | SHR+STZ+Adp+Pio | 1. 165 2. 168 3. 167 4. 171 5. 174 6. 166 |
