# Supplementary material for: Renoprotective and haemodynamic effects of adiponectin and peroxisome proliferator-activated receptor agonist, pioglitazone, in renal vasculature of diabetic Spontaneously hypertensive rats
Source: PLoS One. 2020 Nov 10;15(11):e0229803. doi: 10.1371/journal.pone.0229803 (PMC7654782; doi:10.1371/journal.pone.0229803)
Supplement: S3 File — (DOCX) [file pone.0229803.s006.docx]

| **Agonist** | **Dose** | **WKY+CNT** | **SHR+CNT** | **SHR+STZ** | **SHR+STZ+Pio** | **SHR+STZ+Adp** | **SHR+STZ+Pio+Adp** |
| --- | --- | --- | --- | --- | --- | --- | --- |
| **Noradrenaline** | 25 ng | 1) 25  2) 29  3) 21  4) 29  5) 21  6) 25 | 1) 40  2) 44  3) 36  4) 36  5) 44  6) 40 | 1) 50  2) 53  3) 47  4) 53  5) 47  6) 50 | 1) 32  2) 36  3) 28  4) 28  5) 36  6) 32 | 1)13  2)16  3)10  4)10  5)16  6)13 | 1)9  2)12  3)6  4)12  5)6  6)9 |
|  | 50 ng | 1) 40  2) 44  3) 36  4) 44  5) 36  6) 40 | 1) 60  2) 65  3) 55  4) 65  5) 60  6) 55 | 1) 77  2) 81  3) 73  4) 81  5) 77  6) 73 | 1) 44  2) 41  3) 47  4) 41  5) 45  6) 47 | 1) 22  2) 19  3) 25  4) 19  5) 25  6) 25 | 1) 13  2) 10  3) 16  4) 10  5) 13  6) 16 |
|  | 100 ng | 1) 50  2) 55  3) 45  4) 45  5) 50  6) 55 | 1) 85  2) 91  3) 79  4) 91  5) 85  6) 79 | 1) 98  2) 103  3) 93  4) 98  5) 93  6) 103 | 1) 74  2) 70  3) 78  4) 74  5) 70  6) 78 | 1) 33  2) 41  3) 25  4) 25  5) 41  6) 33 | 1) 20  2) 22  3) 15  4) 15  5) 22  6) 20 |
| **Phenylephrine** | 0.25 µg | 1) 29  2) 33  3) 25  4) 25  5) 33  6) 29 | 1) 39  2) 36  3) 42  4) 36  5) 39  6) 42 | 1) 52  2) 55  3) 49  4) 55  5) 52  6) 49 | 1) 27  2) 24  3) 30  4) 30  5) 27  6) 24 | 1) 18  2) 21  3) 15  4) 18  5) 21  6) 15 | 1) 17  2) 20  3) 17  4) 14  5) 20  6) 14 |
|  | 0.5 µg | 1) 44  2) 40  3) 48  4) 48  5) 40  6) 44 | 1) 80  2) 90  3) 80  4) 75  5) 78  6) 80 | 1) 95  2) 99  3) 91  4) 99  5) 95  6) 91 | 1) 55  2) 59  3) 51  4) 51  5) 55  6) 59 | 1) 27  2) 30  3) 24  4) 24  5) 30  6) 28 | 1) 24  2) 20  3) 28  4) 28  5) 20  6) 24 |
|  | 1 µng | 1) 66  2) 60  3) 74  4) 72  5) 60  6) 64 | 1) 100  2) 90  3) 108  4) 106  5) 94  6) 98 | 1) 115  2) 110  3) 120  4) 119  5) 110  6) 115 | 1) 74  2) 80  3) 69  4) 69  5) 79  6) 75 | 1) 51  2) 60  3) 43  4) 43  5) 59  6) 50 | 1) 38  2) 34  3) 42  4) 42  5) 32  6) 40 |
| **Methoxamine** | 0.5 µg | 1) 17  2) 26  3) 8  4) 8  5) 28  6) 15 | 1) 28  2) 33  3) 25  4) 25  5) 33  6) 28 | 1) 34  2) 31  3) 37  4) 31  5) 37  6) 34 | 1) 12  2) 10  3) 14  4) 14  5) 10  6) 10 | 1) 10  2) 13  3) 7  4) 10  5) 13  6) 7 | 1) 6  2) 3  3) 9  4) 9  5) 3  6) 6 |
|  | 1 µg | 1) 38  2) 46  3) 28  4) 29  5) 46  6) 36 | 1) 55  2) 59  3) 51  4) 51  5) 59  6) 55 | 1) 59  2) 63  3) 55  4) 55  5) 63  6) 59 | 1) 32  2) 35  3) 29  4) 32  5) 30  6) 34 | 1) 23  2) 20  3) 26  4) 26  5)19  6) 22 | 1) 13  2) 16  3) 10  4) 10  5) 13  6) 17 |
|  | 2 µng | 1) 55  2) 62  3) 46  4) 48  5) 62  6) 58 | 1) 72  2) 81  3) 63  4) 62  5) 82  6) 70 | 1) 77  2) 74  3) 82  4) 82  5) 76  6) 72 | 1) 44  2) 41  3) 47  4) 44  5) 41  6) 47 | 1) 30  2) 22  3) 38  4) 36  5) 20  6) 34 | 1) 15  2) 12  3) 18  4) 10  5) 14  6) 18 |
| **Angiotensin II** | 2.5 ng | 1) 19  2) 23  3) 15  4) 15  5) 23  6) 19 | 1) 29  2) 34  3) 24  4) 24  5) 34  6) 29 | 1) 37  2) 40  3) 34  4) 34  5) 40  6) 34 | 1) 21  2) 18  3) 24  4) 24  5) 18  6) 21 | 1) 10  2) 7  3) 13  4) 12  5) 7  6) 10 | 1) 13  2) 10  3) 16  4) 16  5) 10  6) 15 |
|  | 5 ng | 1) 41  2) 46  3) 36  4) 36  5) 46  6) 41 | 1) 55  2) 59  3) 51  4) 51  5) 59  6) 55 | 1) 68  2) 72  3) 64  4) 64  5) 72  6) 68 | 1) 33  2) 36  3) 30  4) 30  5) 35  6) 36 | 1) 23  2) 20  3) 26  4) 26  5) 20  6) 25 | 1) 27  2) 31  3) 23  4) 24  5) 26  6) 30 |
|  | 10 ng | 1) 65  2) 71  3) 59  4) 58  5) 70  6) 68 | 1) 82  2) 89  3) 75  4) 75  5) 90  6) 82 | 1) 89  2) 94  3) 84  4) 95  5) 84  6) 90 | 1) 47  2) 51  3) 43  4) 46  5) 50  6) 44 | 1) 40  2) 42  3) 38  4) 38  5) 42  6) 42 | 1)39  2)35  3)43  4)44  5)35  6)38 |
