# Supplementary material for: Renoprotective and haemodynamic effects of adiponectin and peroxisome proliferator-activated receptor agonist, pioglitazone, in renal vasculature of diabetic Spontaneously hypertensive rats
Source: PLoS One. 2020 Nov 10;15(11):e0229803. doi: 10.1371/journal.pone.0229803 (PMC7654782; doi:10.1371/journal.pone.0229803)
Supplement: Attachment — Submitted filename: Response to Reviewers.pdf [file pone.0229803.s009.pdf]

Reviewer #2: The authors addressed some of the comments but the main core of the questions raised continued unanswered. The renal functions depends in a great deal to water and food intake especially potassium excretion. True hemodynamic parameters such as GFR, filtration fraction, medular blood flow, etc were not used. True markers of renal integrity were not used also neither morphological analysis were performed what difficults interpretation of the data and weakens the conclusions of the authors. The pharmacological study was also not weel designed since 3 drugs in different doses were used in the same animal and no paramenter of compensations (such as sympathetic acitivity, ACE, renin, aldosterone,etc) were measured tu support conclusions. On the top of that no mechanism to explain the claimed benefits of adiponectin and piaglitazone claime renoprotective effect.

Response to Reviewer comments:

We are thankful to the reviewer for acknowledging the comments in the previous version of the manuscripts as "responses to reviewer comments", as we tried our best to address the queries asked raised by the reviewer. However, the remaining points raised by the reviewers are addressed as follows:

The reviewer made a valid point in mentioning that renal functions depend largely on potassium excretion. We agree to this point and understand that dietary intake and excretion of potassium contribute to the genesis of essential hypertension and diabetic hypertensive disease animal models. We had already obtained this parameter in our main study/project entitled: "Haemodynamic effects of adiponectin on PPAR–γ receptors in diabetic and non-diabetic Wistar kyoto and Spontaneously hypertensive rats", but unfortunately couldn't add in this manuscript. Since we use the values for the Na:K ratio, considered as indirect marker of kidney function of aldosterone acting on renal collecting tubules, as renal hemodynamic data, whereas, in this revised version we now have incorporated the values for urinary potassium. However, we didn't measure the food intake as animals were provided food and water ad-libitum, but obtained the values for body weight, water intake and urine flow rate as part of our metabolic indices, keeping in consideration the polydipsia and polyuria characteristics of diabetic disease rat models. Among renal hemodynamic parameters, we measure renal cortical blood perfusion (RCBP) during our acute surgical procedure instead of medullary blood flow rate and GFR. Similarly we obtained urinary creatinine, creatinine clearance, urinary Na & potassium values with baseline and vasoactive RCBP responses to adrenergic agonists to explain and justify the renal integrity and renal vasculature indices in diabetic SHRs, which have been presented in the manuscript to support our study findings. We believe that obtained and presented renal parameters as manuscript data would be quite sufficient to support and interpret our conclusions in terms of renal haemodynamics in diabetic genetic model of hypertensive rats.

This pharmacological study was designed on the basis of our previous laboratory protocols with published data in which three different doses of alpha adrenergic agonists including Na (25.50 and 100 ng), Pe (0.25,0.50 and 1.00 µg), Me (0.25,0.5 and 2.0 µg) and Angiotensin-II (2.5,5.0 and 1.0 ng) as minimum and maximum dosed which could yield vasoactive responses during RCBP using ascending and descending doses fashion to produce a response and then obtained there mean values to present in graphical form. These reference dosed and the studies published earlier using the agonists doses have been quoted in the manuscript reference list and also mentioned in the previous comments, section 5. However, we agree we did not measure morphological analysis and measure the ACE, renin and aldosterone values as compensatory parameters during our study, as it was not part of our experimental settings and project commencement, and we hope to get an excuse in this regard. However, we understand the reviewers concern and will keep in consideration and follow this point as well in our future renal hemodynamic project/studies.

The synergistic renoprotective effects of pioglitazone with adiponectin can be explained on the basis of attenuating renal vascular reactivity to vasoconstrictor agonists probably through eNOs stimulation, which helps in maintaining renal physiology. Literature already supports the fact that pioglitazone, as PPAR-γ, involves adiponectin dependant pathway, and thereby increases its plasma concentration and nitric oxide production. Moreover, adiponectin causes vasodilatory action through No release and shows its renoprotective actions through AMPK-activated pathway expressed in the renal vasculature, besides inhibiting Na+-K+-ATPase activity in renal proximal tubule cells, required for the normal renal functions. The full PPAR-γ agonistic activity of pioglitazone augmented the renoprotective actions of adiponectin, apart from explaining the considerable function of PPAR-γ in control of renal haemodynamics in diabetic SHRs.

Therefore, our study hypothesis is supported and aims that these two drugs avert the angiotensin-II actions (vasoconstrictor agonist) along signaling pathways with a crosstalk relationship between adiponectin receptors and alpha adrenoceptors in the renal vasculature. An explanation to these points have already been discussed in the discussion part of the manuscript.
