# Supplementary material for: Renoprotective and haemodynamic effects of adiponectin and peroxisome proliferator-activated receptor agonist, pioglitazone, in renal vasculature of diabetic Spontaneously hypertensive rats
Source: PLoS One. 2020 Nov 10;15(11):e0229803. doi: 10.1371/journal.pone.0229803 (PMC7654782; doi:10.1371/journal.pone.0229803)
Supplement: Attachment — Submitted filename: comments to authos.docx [file pone.0229803.s007.docx]

Earlier studies have shown that plasma adiponectin concentration is reduced in patients with hypertension and renal dysfunctions. In this manuscript the authors have reported the results of their study exploring the effects of pioglitazone which serves as an agonist of adiponectin and peroxisome proliferator-activated receptor (PPAR-γ), on renal and vascular system in diabetes-induced SHR rats. Their study showed that adiponectin exerts renal protective effects and improves renal hemodynamics through adiponectin receptors and PPAR-γ in diabetic SHR and point to the synergism between adiponectin and pioglitazone.

The study is well designed and executed, the results are interesting, and the paper is well written
