# Supplementary material for: Renoprotective and haemodynamic effects of adiponectin and peroxisome proliferator-activated receptor agonist, pioglitazone, in renal vasculature of diabetic Spontaneously hypertensive rats
Source: PLoS One. 2020 Nov 10;15(11):e0229803. doi: 10.1371/journal.pone.0229803 (PMC7654782; doi:10.1371/journal.pone.0229803)
Supplement: Attachment — Submitted filename: Response to Reviewers.docx [file pone.0229803.s008.docx]

**1. The lack of water and food intake data make it very hard to interpret the results?**

**Response**: The Reviewer has made an important point. Water intake (ml/d) for all the treated groups measured before start of the treatment i.e day 0, then on 8 and day 21 (during treatment period with drugs) and finally on 28, at the end of treatment period, and this data is included has already been included in table 1 of the manuscript. However, we did not observe the food intake as part of our observations, since we were dealing with the renal functions and excretory mechanism involved signifying renoprotective effects of the drugs used in our study.

**2. The lack of real GFR (since diabetic rats face severe proteolysis the use of the creatinine ratio is not a good predictor), filtration fraction and medullar blood flow (which is not auto-regulated and the cortical renal blood flow) also difficult the interpretations of the results;**

**Response:** We agree with the reviewer on this point that GFR could have been determined, but we did not measure it in our experimental protocol, however, the parameters we determined including renal excretory functions with renal cortical blood flow involving vasoconstrictors are sufficient to interpret the results.

**3. Both hypertension and diabetes have increased urinary albumin/creatinine as a hallmark, it would be a better marker than Na/K ratio as a measure of renoprotection. Indeed, no renal marker or histopathological finding support this renoprotective claim of the authors;**

**Response:** The Reviewer has made a valid point. Urinary creatinine with histopathological findings serves as a hallmark and better renoprotective marker in hypertensive and diabetic states. However, we have now added the urinary creatinine values/data observed in our experimental rats used in the study. The data presented in table 3 now shows the values for the urinary creatinine values and also discussed in the discussion part of the manuscript.

**4. The authors discuss about eNOs, Na/KATPase, sympathetic activity, renin levels, aldosterone levels, etc but did not actually measure any of these parameters;**

**Response:** The Reviewer has made an important observation and was something that did concern us. We did not measure these parameters. We only referred to these values parameters to support our findings based on our tested hypothesis and we have provided necessary references for these information. We refer to these parameters to support our observations regarding pioglitazone in combination with adiponectin in possessing renoprotective effects in diabetic SHRs by attenuating renal vascular reactivity probably through eNOS activation, whereas, activation of Na/KATPase modifiy sodium resorption in renal proximal tubule cells of SHR diabetic rats supports our findings in terms of adiponectin that it could prevent the ANG-II actions at various points along signaling pathway to maintaining renal physiology. Similarly renin and aldosterone levels are discussed while justifying the renoprotective characteristics of pioglitazone with adiponectin.

**5. for instance, its very difficult to discuss the data since the authors infuse 4 different agonists both using 3 different doses and we don’t have any data in renal sympathetic activity, renin levels, ACE activity, etc;**

**Response:** The Reviewer has raised an important point. Four different agonists including three alpha adrenergic agonists and ANG-II have been used in ascending and descending order with dose-response relationship to ascertain the renal vasoconstrictor responses in pre-treated diabetic SHRs with PPAR- agonist and adiponectin in singly and combination. The doses used for all these agonists have already been confirmed in our lab studies earlier (Abdullah et al., 2009, Armenia et al., 2004, S. Afzal et al., 2016, and Khan et al., 2009 with references quote in the literature cited at number 20, 23, 25, and 33. However, we did not measure the renal sympathetic activity with renin levels and ACE activity as these parameters were not included in our experimental settings.

**6. UNaV, FENa are not hemodynamic parameters and should not be considered as hemodynamic data in this manuscript;**

**Response:** We use the term “Renal haemodynamics” as the parameters involve the physiological and metabolic indices along renal excretory parameters involving Creatinine clearance, Urine creatinine, Urinary Sodium concentration, Absolute urinary sodium excretion, Fractional sodium excretion and Urinary sodium to potassium ratio.

**7. The authors claim that adiponectin induces natriuresis and diuresis but this data is not shown in the present manuscript;**

**Response:** The Reviewer observation been addressed and to support our data we add the values for Urinary sodium concentration in table 3, as we already obtained the values for urinary sodium concentration but unfortunately couldn’t add in our previous manuscript version discussed in discussion part, whereas, the values for UFR (mL/min/100 g) already in table 1 and both of these values can be considered as factor causing diuresis in our experimental findings.

**Minor comments:
1. 2 ml of blood sampling seems too excessive, it is there a good reason for such a high volume of blood taken from this already critic diabetic animals??**

**Response:** The Reviewer is quite correct in asking for withdrawing 2 ml of the blood samples which is too excessive. This data shown in this study is a part of the whole project entitled “haemodynamic effects of adiponectin on PPAR–γ receptors in diabetic and non-diabetic Wistar kyoto and Spontaneously hypertensive rats”, which also observed the antioxidant and systemic haemodynamic parameters as part of the study. The reason to withdraw 2 ml of blood samples was to measure the plasma concentration of various *in-vitro* and *in-vivo* parameters including glycaemic, lipid profile antioxidant and systemic hemodynamic data observations which require at least a sufficient amount of blood samples, keeping in view the statistical and biochemical analysis of the tests using various biochemical kits to measure the above mentioned parameters.

**2.NA/PE/ME are cited without any previous description. Whats the rationale to use these drugs all together in one animal. Why not to choose only one or at most two?**

The Reviewer has made an important point. The alpha(1) and alpha(2) adrenoceptor subtypes were previously defined pharmacologically by functional and binding studies, and later they were also isolated and identified using cloning methods (Civantos Calzada and Aleixandre de Artinano 2001), Sattar, M. A. and E. J. Johns (1996, 1994). We use alpha adrenergic agonists (NA, PE and ME) acting on subset of alpha adrenoceptor -1-adrenoceptors (α1A, α1B and α1D). Noradrenaline acts on α1 and α2-adrenoceptors; whereas PE acts selectively to activate α1A-, α1B-, and α1D-adrenoceptor subtypes and ME activates only α1A adrenoceptors-subtype. A sentence to this effect has already been explained in methodology section (p 04, Lines 16-20).

Furthermore, our study also test the hypothesis that PPAR-γ a cross-talk relationship exists between PPAR-γ, and alpha adrenoceptor subtypes (α1A-, α1B- and α1D) in the renal vasculature of diabetic model of SHRs, which allow us to use 3 subtypes of alpha adrenoceptors.

**3. 3 days of acclimatization period seems to be a short period of adaptation and in this condition food and water intake should have been reported, especially because potassium excretions is very sensitive to diet.**

**Response:** For this study we follow the ARRIVE and Animal Ethics Committee, Universiti Sains Malaysia, (AECUSM), Malaysia guidelines. We kept the animals in the animal transit room for acclimatization purpose, and during this period of they were provided with food and water *ad-libitum* using Commercial rat chow feed and fresh plain water with frequency of monitoring the animal’s health was twice to rule out any abnormality or disease condition during the acclimatization period and also before the experimental procedures. The water intake data has been shown in table 1, whereas, we did not measure the food intake during acclimatization and experiment as it was not the part of our study protocols.
